# Lead Sources in Human Diet in Greenland

**DOI:** 10.1289/ehp.7083

**Published:** 2004-07-21

**Authors:** Peter Bjerregaard, Poul Johansen, Gert Mulvad, Henning Sloth Pedersen, Jens C. Hansen

**Affiliations:** ^1^National Institute of Public Health, Copenhagen, Denmark; ^2^National Environmental Research Institute, Roskilde, Denmark; ^3^Primary Health Care Center, Nuuk, Greenland; ^4^Centre for Arctic Environmental Medicine, University of Aarhus, Aarhus, Denmark

**Keywords:** diet, Greenland, Inuit, lead, lead shot, sea birds

## Abstract

Although blood lead levels have declined in Greenland, they are still elevated despite the fact that lead levels in the Greenland environment are very low. Fragments of lead shot in game birds have been suggested as an important source of dietary exposure, and meals of sea birds, particularly eider, contain high concentrations of lead. In a cross-sectional population survey in Greenland in 1993–1994, blood lead adjusted for age and sex was found to be associated with the reported consumption of sea birds. Participants reporting less than weekly intake of sea birds had blood lead concentrations of approximately 75 μg/L, whereas those who reported eating sea birds several times a week had concentrations of approximately 110 μg/L, and those who reported daily intake had concentrations of 170 μg/L (*p* = 0.01). Blood lead was not associated with dietary exposure to other local or imported food items.

Lead has been recognized as a poison for millennia and has recently been the focus of public health regulations in most of the developed world. Consequently, fatalities and symptomatic lead poisoning have declined dramatically during the latest decades and are continuing to decline (Kaufmann et al. 2003). In recognition of the particular sensitivity of the developing brain to lead effects, much of this legislation has addressed the prevention of childhood lead poisoning. Of particular importance is the accumulation of data suggesting that there are toxicologic effects in children at low levels of exposure (Winneke et al. 1996). Lanphear et al. (2000) found that deficits in cognitive and academic skills in children 6–16 years of age were associated with lead exposure at blood lead concentrations < 50 μg/L. In addition, evidence shows that certain genetic and environmental factors can increase the detrimental effects of lead on neural development (Lidsky and Schneider 2003; Long et al. 2002). Long-term deficits in cognitive function are the principal effects of lead exposure in children and can be modeled in experimental animals (Guilarte et al. 2003). During the past two decades, the proportion of U.S. children who have blood lead concentrations ≥100 μg/L declined by > 80% after the elimination of leaded gasoline and lead solder from canned foods and a ban on leaded paint used in housing (Lanphear et al. 2003). Furthermore, lead is associated with elevated blood pressure and cardiovascular mortality. The association is weak, but there is a small dose response through the range of blood concentrations ≥350 μg/L (Hertz-Picciotto and Croft 1993).

Blood lead levels in samples collected before 1980 from the indigenous (Inuit) adult population of Greenland were found to be similar to those of populations in western European cities, where leaded gasoline was the main source (Hansen 1981; Hansen et al. 1983). Because local sources of lead in Greenland were considered unlikely to be significant, the cause of the unexpected high levels was proposed to be long-distance atmospheric transport of lead particles in combination with an increased intestinal absorption due to a diet low in calcium and rich in iron and protein (Hansen 1988; Milman et al. 1994). This assumption was supported by the finding that lead was being transported and deposited in the Greenland ice cap from remote sources (Boutran et al. 1995). Further support for the assumption is that in samples collected after 1980, blood lead concentrations have gradually declined in parallel with the reduced use of leaded gasoline on a global scale (Hansen et al. 1991). However, blood lead levels in Greenland appear still to be elevated compared with those in other Arctic regions and Scandinavia. Levels among Greenland Inuit mothers (geometric means, 31–50 μg/L) were found to be similar to the moderately increased levels among some of the Canadian Inuit [Arctic Monitoring and Assessment Programme (AMAP) 2003; Bjerregaard and Hansen 2000].

In Canada, elevated levels of blood lead in children were proposed to be caused by the consumption of birds containing lead shot (Smith and Rea 1995), and high levels of lead were subsequently detected in bird meat (Scheuhammer et al. 1998). Later research on lead isotope signatures in the Canadian Arctic has indicated that elevated blood lead levels were likely caused by the use of lead shot and thus its presence in the wild game consumed (Dewailly et al 2000). High levels of lead in tissue were also found in Greenland by Johansen et al. (2001), who concluded that birds killed by lead shot probably is the most important source of lead in the diet of many people in Greenland. In 2004, lead shot is still commonly used in Greenland for hunting, and a recent study has shown that game birds contain even higher concentrations of lead than found earlier (Johansen et al. 2004). The lead shots are fragmented on impact, and the meat of the birds is consequently contaminated by microscopic particles of lead. These researchers concluded that in some cases safe limits of lead intake by humans would be largely exceeded.

The purpose of the study reported here was to analyze the association of blood lead with the consumption of traditional and store-bought food items in a cross-sectional population survey in Greenland conducted in 1993–1994. Since then, there have been no changes in the use of lead shot for hunting. Also, traditional food (fish, birds, and mammals) still constitutes an important part of the diet in Greenland, although with large variation among regions and individuals.

## Materials and Methods

The total population of Greenland was 55,000 in 1993, of whom 86% were born in Greenland (a proxy measure of Inuit ethnicity). Genetically, the Greenlanders are Inuit with a substantial admixture of European genes. They are historically, culturally, and genetically closely related to the Inuit of Canada and the Inupiat of Alaska and speak mutually intelligible dialects of the same language. The population is scattered along the coastline in 17 towns and 60 villages, most of which are situated on the west coast between the 60th and the 75th parallels. Only 19% of the 18- to 59-year-old male Greenlanders rely on hunting or fishing for a living, but subsistence hunting as a supplement to a paid job is common (Bjerregaard et al. 1995).

During 1993–1994, a sample of the inhabitants in Greenland, selected at random from the central population register, was asked to participate in a health interview survey. From the 1,728 participants, a subsample of Greenlanders (Inuit) from three towns and four villages on the west coast of Greenland were selected at random for the present study (*n* = 228). Among these, the interview was supplemented with a clinical examination and blood sampling. The subsample consisted of fewer men than the total Inuit population of Greenland (42 and 51%, respectively), and the 18- to 34-year-old age group was especially underrepresented.

The dietary questionnaire was developed specifically for this study because there are no standardized questionnaires on traditional Greenlandic food available. The questionnaire was of the food frequency type and included questions on traditional Greenlandic food and certain categories of imported food. Among 17 food categories, four included traditional Greenlandic food items: seal, whale, wild fowl (the vast majority of which are sea birds), and fish. The frequency categories were daily, 4–6 times a week, 1–3 times a week, 2–3 times a month, once a month or less, and rarely.

Blood samples were obtained after overnight fasting. The blood was separated, frozen at −20°C, and shipped to Denmark, where the samples were analyzed for lead by atomic absorption spectrometry at the National Environmental Research Institute of Denmark (Department of Arctic Environment, Roskilde, Denmark) (Asmund and Cleemann 2000). The detection limit of the method was calculated to be 6 μg/L.

**Figure 1:**
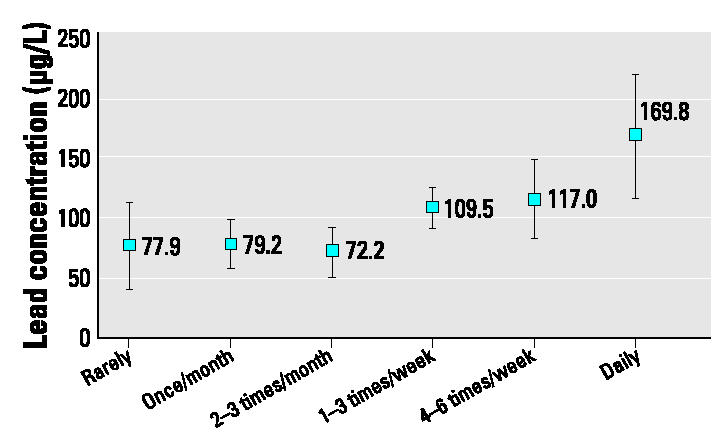
Blood lead concentration according to reported consumption of sea birds adjusted for age and sex (arithmetic means with 95% confidence intervals): Greenland 1993–1994 (*n* = 161).

Data analysis was performed with SPSS/ Windows (version 11.5; SPSS Inc., Chicago, IL, USA). *p*-Values were calculated by analysis of variance from log-transformed blood lead values. The association between several dietary variables and blood lead was explored in a general linear model with control for age and sex (Figure 1). Nonsignificant variables were removed by backward elimination.

Ethical approval was obtained from the Commission for Scientific Research in Greenland. Informed consent was obtained verbally.

## Results

Dietary information was obtained from 222 participants, and blood lead analysis was carried out on 162 of these (73%). The sub-sample consisted of 67 men (median age, 43 years; range, 23–77 years) and 94 women (median age, 39 years; range, 20–78 years). After the removal of one outlier with a lead concentration of 1.0 μg/L, the mean ± SD concentration of blood lead in this population was 94.4 ± 69.6 μg/L (range, 7–351 μg/L). Lead concentrations increased significantly with age (*p* < 0.001) and were higher among men than among women (103 and 88 μg/L; *p* = 0.05) but were not significantly associated with body mass index, smoking, or consumption of alcohol.

Blood lead concentrations were not associated with the reported consumption of any of the imported food items but were associated with several local marine food items, particularly sea birds (Table 1). The dietary variables were all closely associated with each other and with both age and sex. Bivariate correlations between the dietary variables ranged from 0.29 between sea birds and whale meat to 0.56 between sea birds and fish (*p* < 0.01 for all correlations). In a multivariate analysis age, sex, and consumption of sea birds were retained in the model, whereas the other dietary variables were not. Adjusted for these covariates, consumption of sea birds was still significantly associated with blood lead (*p* = 0.01; Figure 1).

A linear regression analysis of blood lead as a predictor for blood pressure with age and body mass index as covariates did not show any association between lead and blood pressure.

## Discussion

Our results indicate that Greenlanders who report consuming sea birds several times a week have a blood lead level > 50% higher than those who report eating sea birds only a few times per month or less often. In combination with a study conducted in 2003 (Johansen et al. 2004) of lead concentrations in breast meat of eider and murre—the two most consumed species of birds in Greenland—this strongly indicates a causal relationship between the consumption of sea birds and blood lead concentrations.

The association between blood lead and the consumption of fish and whale is not considered causal, because lead concentrations in fish and whales (and other local food) are very low (Johansen et al. 2000). The cause is rather that people who eat many birds also eat much fish and whale.

We made a rough estimate of the lead intake from birds in Greenland. The dominating species in Greenland bird hunting are the thick-billed murre and the common eider. From 1994 to 1999, the annual reported hunt ranged from 187,685 to 254,728 murres and from 72,109 to 83,810 eiders (Anonymous 2001). Johansen et al. (2004) calculated the mean lead intake to be 146 μg lead from one murre meal and 1,220 μg lead from one eider meal. If we assume that murres are eaten three times as often as eiders (as could be indicated by the hunting figures), the lead intake from “an average bird meal” will be 0.25 × 1,220 μg lead + 0.75 × 146 μg lead = 425 μg lead.

The Food and Agriculture Organization (FAO)/World Health Organization (WHO) (1993) has established a provisional tolerable weekly intake (PTWI) equivalent to 1,500 μg lead/week for a person weighing 60 kg (25 μg/ kg/day). This recommendation was maintained at the meeting of the Joint WHO/FAO Expert Committee of Food Additives in 1999 (WHO 2000). The calculation shows that the PTWI of 1,500 μg for a 60-kg person will be exceeded when eating four bird meals or more per week. Birds are, however, eaten seasonally, and the reported average consumption of seabirds cannot be meaningfully compared with blood lead levels at one point in time. Further studies where bird consumers are followed before, during, and after the bird hunting season are needed to establish the association between consumption of birds and blood lead concentrations as well as peak concentrations.

The lead intake from other dietary sources is estimated to be significantly lower than that from tissue contaminated with lead shot. The lead intake from the traditional diet in Greenland has been estimated to be only 15 μg per person per week (Johansen et al. 2000), and the lead intake from imported food is also considered to be at a low level because food (with low lead levels) imported from Denmark dominates the market in Greenland. In Denmark the mean lead intake from food is estimated to be 126 μg/week (Larsen et al. 2002).

Earlier theories that elevated blood levels in Greenland were caused by long-range transport of lead, mainly from leaded gasoline (Hansen 1988; Milman et al. 1994), must be rejected based on this study and others pointing to lead shot as the main source in Greenland, Canada, and Russia (Hanning et al. 2003; Johansen et al. 2004; Odland et al. 1999; Scheuhammer et al. 1998; Smith and Rea 1995). This is supported by the finding that the atmospheric lead concentration at Station Nord in remote northeast Greenland showed no change from 1990 to 2001 (Heidam et al. 2004), whereas it has declined by about a factor of 10 in Copenhagen (Kemp and Palmgren 2002). Also, the lead concentration in the air in Greenland is approximately 10 times lower than in Denmark, whereas the blood lead concentration is lower in Denmark than in Greenland (Nielsen et al. 1998). Considering that the lead concentration would be diluted during long-range transport, it seems unlikely that such lead could be significant as a direct source when breathing the air in Greenland.

However, blood lead levels in Greenland have declined over the past 20 years (AMAP 2003; Hansen 1981; Hansen et al. 1983). It is possible that the decline has been caused by a lower consumption of birds. Another possible explanation is that leaded gasoline was phased out during the 1990s and is no longer used in Greenland; this may have been a significant local source earlier, both from combustion and from handling of gasoline.

## Figures and Tables

**Table 1:** Blood lead concentrations (μg/L) according to diet (arithmetic means): Greenland 1993–1994 (*n* = 161).

Reported frequency of consumption	Seal	Whale	Sea birds	Fish
Rarely (*n* = 12)	81	98	74	—
Once a month (*n* = 39)	86	71	71	92
2–3 times per month (*n* = 36)	74	97	70	60
1–3 times per week (*n* = 53)	96	112	114	96
4–6 times per week (*n* = 15)	93	102	127	109
Daily (*n* = 6)	131	169	181	139
*p*-Values	*p* = 0.22	*p* = 0.04	*p* < 0.001	*p* = 0.001

*p*-Values were calculated from log-transformed concentrations.
